# Glucose and lipoprotein biomarkers and breast cancer severity using data from the Swedish AMORIS cohort

**DOI:** 10.1186/s12885-017-3232-6

**Published:** 2017-04-04

**Authors:** Jennifer C Melvin, Hans Garmo, Lars Holmberg, Niklas Hammar, Göran Walldius, Ingmar Jungner, Mats Lambe, Mieke Van Hemelrijck

**Affiliations:** 1 King’s College London, Division of Cancer Studies, Translational Oncology and Urology Research (TOUR), Research Oncology, Guy’s Hospital, 3rd Floor, Bermondsey Wing, London, SE1 9RT UK; 2 Regional Cancer Centre, Uppsala/Ӧrebro, Uppsala, Sweden; 3 Unit of Epidemiology, Insitute of Environmental Medicine, Karolinska Institutet, Stockholm, Sweden; 4 AstraZeneca R&D, Mölndal, Sweden; 5 Department of Epidemiology, Institute of Environmental Medicine, Karolinska Institutet, Stockholm, Sweden; 6 Department of Medicine, Clinical Epidemiological Unit, Karolinska Institutet and CALAB Research, Stockholm, Sweden; 7 Department of Medical Epidemiology and Biostatistics, Karolinska Institutet, Stockholm, Sweden

**Keywords:** Breast cancer (BC), Glucose., Triglycerides., Total cholesterol., HDL cholesterol., LDL cholesterol,, Apolipoprotein A-I,, Apolipoprotein B,, Severity,, Prognosis.

## Abstract

**Background:**

The lipid and glucose metabolisms are postulated as possible intermediary mechanisms in linking obesity and breast cancer (BC). Links between serum lipid and glucose biomarkers and BC risk has been observed in the Swedish Apolipoprotein MORtality RISk (AMORIS) cohort. We conducted a follow-up analysis including information on tumour characteristics.

**Methods:**

One thousand eight hundred twenty-four women diagnosed with BC, with serum biomarker levels of glucose, triglycerides, cholesterol (total, HDL, and LDL), and apolipoproteins A-1 and B recorded in a routine health check at baseline were included. BC severity was split into categories (good, moderate, and poor prognosis) based on ER status, TNM stage, and age at diagnosis. Proportional odds models were used to obtain odds ratios (ORs) and 95% confidence intervals (CI), with the interval time between baseline measurement and BC diagnosis accounted for.

**Results:**

Serum glucose and the ApoB/ApoA-1 ratio showed a non-statistically significant positive association with BC severity (proportional OR: 1.25 (95%CI: 0.92–1.70) for glucose (</≥ 5.60 mmol/L) and 1.31 (95%CI: 0.97–1.76) for ApoB/A-1 ratio (</≥ 1). The proportion of severe and moderate BC was modestly greater across all abnormal serum biomarker groups.

**Conclusions:**

Despite the size and detail of data in AMORIS, we only found a modest positive association between serum levels of glucose, apoB/ApoA-1 and BC severity, suggesting that these factors are not the main players in linking obesity and BC aggressiveness.

**Electronic supplementary material:**

The online version of this article (doi:10.1186/s12885-017-3232-6) contains supplementary material, which is available to authorized users.

## Background

Epidemiological evidence suggests a positive link between obesity, overweight, and risk, progression, and severity of breast cancer (BC) [9, 31]. Moreover, an association between the metabolic syndrome (MetS) and a worse BC prognosis has been reported [10]. MetS is defined by a combination of at least three of the following metabolic risks: visceral obesity, elevated serum triglycerides, reduced high-density lipoprotein cholesterol, raised blood pressure and raised serum glucose [6]. Despite evidence from in vitro research [8, 19, 24], the specific underlying mechanisms underpinning the link between obesity, MetS, and BC progression have yet to be fully elucidated and epidemiological findings remain contradicting [25, 32, 34, 41].

One mechanism suggested for this association is increased oestrogen levels -- sourced from the fat in adipose tissue -- which are synthesised from cholesterol [17]. Leptin, insulin-like growth factors (IGF), and the lipid and glucose metabolisms have also been postulated as possible intermediate mechanisms responsible for associations between obesity and BC risk [7, 33]. A positive association between triglycerides and BC risk has been observed previously [13]. Moreover, the unfavourable hormonal profile (e.g., elevated insulin, oestrogen, or leptin) associated with low levels of high-density lipoprotein (HDL) is thought to increase BC risk [22]. There is also evidence that higher levels of low-density lipoprotein (LDL) at time of BC diagnosis are indicative of poor prognosis [41]*.*

Based on data in the AMORIS cohort, a large Swedish database with information on over 800,000 men and women, we have previously identified some evidence that abnormal serum lipid profiles, measured about 8 years prior to diagnosis, may be involved in the risk of developing BC [35]. Furthermore, a second study within the same population indicated that increased glucose levels, even those below the diabetic threshold, are positively associated with risk of postmenopausal BC [33]. Here, we further investigated these observations by also taking into consideration information on tumour characteristics and classifying BC into categories of severity.

## Methods

### Study Population & Data Collection

The Apolipoprotein Mortality Risk (AMORIS) Study has been described in detail elsewhere (11,12). Briefly, the recently updated AMORIS database comprises 812,073 individuals with blood samples sent for laboratory testing to the Central Automation Laboratory (CALAB) in Stockholm, Sweden, during the period 1985 to 1996. Individuals recruited were mainly from the greater Stockholm area, and either healthy and having laboratory testing as a part of general check-up, or outpatients referred for laboratory testing. None of the participants were inpatients at the time the samples were analysed. In the AMORIS study, the CALAB database was linked to Swedish national registries such as the Swedish National Cancer Register, the Hospital Discharge Register, the Cause of Death Register, the consecutive Swedish Censuses during 1970–1990, and the National Register of Emigration using the Swedish 10-digit personal identity number, providing complete follow-up information until 31 December 2011. For the current study, we specifically made use of the linkage between AMORIS and the Quality Register for Breast Cancer [18, 26–28, 46–48, 51, 52].

The main purpose of the Regional Quality Register of Breast Cancer in the Stockholm healthcare region is to monitor the quality of care based on regional or national guidelines for BC management. The register includes individual information reported continuously from the clinicians on date of diagnosis, detection mode, pathological tumour-stage, tumour characteristics and primary surgical and oncological treatment for all newly diagnosed BC patients. The database is continuously updated against the National Population Register to assess current vital status of the registered patients. The Regional Quality Register of Breast Cancer in the Stockholm healthcare region started in 1976, and has a 97% coverage, following validation against the records of the mandatory Swedish National Cancer Register.

Additional file 1: Figure S1 illustrates the cohort selection for this study. From the subgroup of 1824 women aged 20 years or older diagnosed with invasive BC who had their serum levels of triglyceride (TG), total cholesterol (TC), glucose, high-density lipoprotein (HDL), low-density lipoprotein (LDL) (in mmol/L), apolipoprotein A-I (ApoA-1), and apolipoprotein B (ApoB) measured (in g/L) at least 3 months prior to diagnosis, we selected all 1499 women whose diagnosis of BC was also registered in the Clinical Quality Register for Breast Cancer. All serum levels were dichotomised according to their medical cut-offs. For HDL, LDL, TC/HDL, LDL/HDL, and ApoB/ApoA-1 these cut-offs were based on the values used in cardiovascular disease prevention (1.03 mmol/l, 4.10 mmol/l, 5.00, 3.50, and 1.00 respectively [20, 29, 36]. Levels of TG, TC and glucose were dichotomised based on the National Cholesterol Education Programme, WHO Diabetes guidelines and the American Heart Association/National Heart, Lung and Blood Institute (1.71, 6.50, and 5.60 mmol/l, respectively) [5, 20, 23]. For each woman, we also calculated the interval time between the time from blood analyses and date of BC diagnosis

TC and TG were measured enzymatically, whereas ApoA-1 and B were measured by immunoturbidimentric methods, with levels standardised according to the World Health Organisation International Federation of Clinical Chemists protocols [28, 29]. Glucose was measured enzymatically with a glucose oxidase/peroxidase method. LDL and HDL concentrations were calculated and validated, and the procedures used have been described in detail elsewhere [51]. All methods were fully automated with automatic calibration and performed at one accredited laboratory [28].

This study complied with the Declaration of Helsinki, and the ethics review board of the Karolinska Institute approved the study (diary number: 2010/1047–31/1).

### Data analysis

**Fig. 1:**
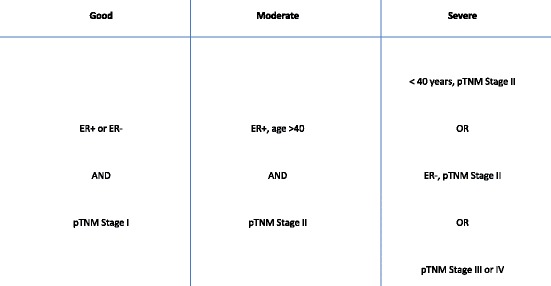
Information from the Regional Quality Register of Breast Cancer was used to categorise breast cancer severity into good, moderate, and sever prognosis

BC was classified into three severity groups. The part of the database linked to the Regional Quality Register of Breast Cancer in the Stockholm healthcare region holds information on ER status, age at diagnosis, and TMN stage, which was used to categorise BC severity into good, moderate, and severe prognosis (Fig. 1). To validate these categories of severity we assessed how they predicted survival (i.e., are those women classified as ‘severe’ actually more likely to have a poorer prognosis relative to the other two severity categories) for all BC patients in the entire AMORIS cohort (*n* = 12,537) using a Kaplan Meier analysis. The results showed a good validity (Additional file 2: Figure S2).

Proportional odds ratios were used to investigate associations between medical cut-offs and ratios of serum lipid and glucose components and BC severity, as the latter is an ordinal categorical outcome measurement. Due to lack of information, subgroup analyses could not be done for HER2 status. When not strongly correlated with the exposure variable of interest, all models were adjusted for glucose, TG and TC levels, as well as age, fasting status, parity (defined as the number of live births), Charlson Co-morbidity Index (CCI), socio-economic status (SES), and tumour characteristics. CCI was calculated through information derived from the National Patient Register. CCI considers 19 diseases, with each disease category assigned a weight [14, 30]. The sum of an individual’s weights was used to create a score, resulting in four co-morbidity levels ranging from no co-morbidity to severe co-morbidity (0, 1, 2, and ≥3). No data was available on menopausal status, but age was included in the severity criteria as a proxy. It was not possible to include information on BMI as this was missing for the majority of women in the AMORIS cohort [35].

In a further analysis we stratified by interval time between baseline measurement and BC diagnosis to investigate the possibility of pre-diagnosed disease influencing measurement (reverse causality). If the proportional odds assumption did not hold, we performed logistic regression analyses comparing good versus moderate/poor and good/moderate versus poor, which is consistent with the comparisons made in a proportional odds model [3].

Finally, Chi-square tests were used to investigate the difference in proportions of BC severity at the time of diagnosis, based on medical cut-offs for each of the biomarkers measured at baseline. A figure was then created which provided a visual comparison of the differing proportions of BC severity at time of diagnosis. All analyses were conducted with Statistical Analysis Systems (SAS) release 9.3 (SAS Institute, Cary, NC) and R version 2.15.13 (R Foundation for Statistical Computing, Vienna, Austria).

## Results

The mean interval time from baseline measurement to BC diagnosis was 11.6 years (±6.5 SD) in all 1824 women diagnosed with BC. Of these, 1499 had sufficient information on tumour characteristics recorded to allow definition of severity status (mean interval time 11.7 years (±6.5)). Because the majority of the measurements recorded in AMORIS were taken as part of routine health check-ups, the bulk of the population (approximately 90%) was gainfully employed (Table 1). Women with severe BC had the highest proportion of BC-specific deaths (>28%). There were no apparent differences in parity, socioeconomic status or serum biomarker distribution (Tables 1 and 2) between BC severity categories.

**Table 1:** Descriptive characteristics by breast cancer severity, including women from the AMORIS database 1989–2011

	Any BC(*N* = 1824)	All BC w/severity (*N* = 1411)	Good (*N* = 900)	Moderate (*N* = 327)	Severe (*N* = 184)
	N (%)	N(%)	N(%)	N(%)	N(%)
Age at Baseline (years)					
≤ 49	810 (44.41)	632 (44.79)	405 (45.00)	133 (40.67)	94 (51.09)
50–59	572 (31.36)	459 (32.43)	310 (34.44)	93 (28.44)	56 (30.43)
60–69	308 (16.89)	235 (16.65)	140 (15.56)	69 (21.10)	26 (14.13)
≥ 70	134 (7.35)	85 (6.02)	45 (5.00)	32 (9.79)	8 (4.35)
Mean Interval (years)					
Time from baseline to BC (SD)	11.56 (6.46)	11.70 (6.52)	11.71 (6.42)	12.67 (6.85)	9.91 (6.06)
Parity					
Yes	1389 (76.15)	1072 (75.97)	682 (75.78)	246 (75.23)	144 (78.26)
No	435 (23.85)	339 (24.03)	218 (24.22)	81 (24.77)	40 (21.74)
Socioeconomic Status					
High	747 (40.95)	594 (42.10)	397 (44.11)	128 (39.14)	69 (37.50)
Low	895 (49.07)	689 (48.83)	432 (48.00)	160 (48.93)	97 (52.72)
Unclassified/Missing	182 (9,98)	128 (9.07)	71 (7.89)	39 (11.93)	18 (9.78)
Fasting Status					
Fasting	1186 (65.02)	888 (62.93)	557 (61.89)	216 (66.06)	115 (62.50)
Non-fasting	256 (14.04)	204 (14.46)	121 (13.44)	43 (13.15)	40 (21.74)
Unknown	382 (20.94)	319 (22.61)	222 (24.67)	68 (20.80)	29 (15.76)
Tumour Side					
Left	759 (50.63)	701 (49.68)	440 (48.49)	168 (47.56)	93 (50.54)
Right	740 (49.37)	674 (47.77)	428 (47.56)	159 (48.62)	87 (47.28)
Missing	325 (17.82)	36 (2.55)	32 (3.56)	0 (0.00)	4 (11.11)
Invasive Grade^a^					
Grade 1	124 (6.80)	113 (8.01)	98 (10.89)	14 (4.28)	1 (0.54)
Grade 2	296 (16.23)	280 (19.84)	178 (19.78)	93 (28.44)	9 (4.89)
Grade 3	170 (9.32)	161 (11.41)	70 (7.78)	56 (17.13)	35 (19.02)
Missing	1234 (67.65)	857 (60.74)	544 (61.56)	164 (50.15)	139 (75.54)
T-Stage					
T0: no evidence of primary tumour	23 (1.26)	21 (1.49)	17 (1.89)	1 (0.31)	3 (1.62)
T1: ≤ 2 cm	1018 (55.81)	966 (68.46)	883 (98.11)	56 (17.13)	27 (14.67)
T2: 2-5 cm	471 (25.82)	365 (25.87)	0 (0.00)	256 (78.29)	109 (59.24)
T3: > 5 cm	36 (1.97)	21 (1.49)	0 (0.00)	14 (4.28)	7 (3.80)
T4: tumour of any size with extension to the chest wall and/or skin	41 (2.25)	21 (1.49)	0 (0.00)	0 (0.00)	38 (20.65)
TX: primary tumour cannot be assessed	235 (12.88)	38 (2.69)	0 (0.00)	0 (0.00)	0 (0.00)
N-Stage					
N0: negative	1332 (73.03)	1233 (87.38)	900 (100.00)	227 (69.42)	106 (57.61)
N1: 1–3	226 (12.39)	167 (11.84)	0 (0.00)	100 (30.58)	67 (36.41)
N2: 4–9	8 (0.44)	5 (0.35)	0 (0.00)	0 (0.00)	5 (2.72)
N3: ≥ 19	7 (0.38)	6 (0.43)	0 (0.00)	0 (0.00)	6 (3.26)
Nx: nodes cannot be assessed	251 (13.76)	0 (0.00)	0 (0.00)	0 (0.00)	0 (0.00)
M-Stage					
M0: no distant spread	1508 (82.68)	1390 (98.51)	900 (100.00)	327 (100.00)	163 (88.59)
M1: spread to distant organs	22 (1.21)	21 (1.49)	0 (0.00)	0 (0.00)	21 (11.41)
Mx: presence of metastasis cannot be assessed	294 (16.12)	0 (0.00)	0 (0.00)	0 (0.00)	0 (0.00)
Oestrogen Receptor Status					
Positive	1013 (55.54)	974 (69.03)	626 (69.56)	327 (100.00)	21 (11.41)
Negative	220 (12.06)	213 (15.10)	87 (9.67)	0 (0.00)	126 (68.48)
Missing	591 (32.40)	224 (15.88)	187 (20.78)	0 (0.00)	37 (20.11)
Progesterone Receptor Status					
Positive	839 (46.00)	811 (57.48)	531 (59.00)	246 (75.23)	34 (18.48)
Negative	379 (20.78)	361 (25.58)	172 (19.11)	77 (23.55)	112 (60.87)
Missing	606 (33.22)	239 (16.94)	197 (21.89)	4 (1.22)	38 (20.65)
HER2-status					
Positive	38 (2.08)	32 (2.27)	13 (1.44)	13 (3.98)	6 (3.26)
Negative	289 (15.84)	264 (18.71)	172 (19.11)	71 (21.71)	21 (11.41)
Missing	1497 (82.07)	1115 (79.02)	715 (79.44)	243 (74.31)	157 (85.33)
CCI					
0	1681 (92.16)	1306 (92.56)	834 (92.67)	302 (92.35)	170 (92.39)
1	70 (3.84)	54 (3.83)	32 (3.56)	14 (4.28)	8 (4.35)
2	54 (2.96)	35 (2.48)	24 (2.67)	8 (2.45)	3 (1.63)
3+	19 (1.04)	16 (1.13)	10 (1.11)	3 (0.92)	3 (1.63)
Dead					
BC-Death	245 (13.43)	371 (26.29)	56 (6.22)	48 (14.68)	60 (32.61)

^a^Only diagnoses taken from the Breast Cancer Quality Register will have information available

**Table 2:** Serum lipids, lipoproteins, and glucose by breast cancer severity, including women from the AMORIS cohort 1989–2011

	Any BC (*N* = 1824)	All BC w/severity (*N* = 1411)	Good (*N* = 900)	Moderate (*N* = 327)	Severe (*N* = 184)
	N(%)	N(%)	N(%)	N(%)	N(%)
Total Cholesterol (mmol/l)					
< 6.50	1333 (73.08)	1040 (73.71)	671 (74.56)	231 (70.64)	138 (75.00)
≥ 6.50	491 (26.92)	371 (26.29)	229 (25.44)	96 (29.36)	46 (25.00)
Triglycerides (mmol/l)					
< 1.71	1592 (87.28)	1237 (87.67)	793 (88.11)	283 (86.54)	161 (87.50)
≥ 1.71	232 (12.72)	174 (12.33)	107 (11.89)	44 (13.46)	23 (12.50)
Glucose (mmol/l)					
< 5.60	1590 (87.17)	1231 (87.24)	797 (88.56)	273 (83.49)	161 (87.50)
≥ 5.60	234 (12.83)	180 (12.76)	103 (11.44)	54 (16.51)	23 (12.50)
HDL (mmol/l)					
≥ 1.03	1763 (96.66)	1364 (96.67)	872 (96.89)	313 (95.72)	179 (97.28)
< 1.03	61 (3.34)	47 (3.33)	28 (3.11)	14 (4.28)	5 (2.72)
LDL (mmol/l)					
< 4.10	1325 (72.64)	1032 (73.14)	665 (73.89)	231 (70.64)	136 (73.91)
≥ 4.10	499 (27.36)	379 (26.86)	235 (26.11)	96 (29.36)	48 (26.09)
Apolipoprotein A (g/L)					
≥ 1.05	1811 (99.29)	1402 (99.36)	896 (99.56)	324 (99.08)	182 (98.91)
< 1.05	13 (0.71)	9 (0.64)	4 (0.44)	3 (0.92)	2 (1.09)
Apoliporotein B (g/L)					
< 1.50	1556 (85.31)	1208 (85.61)	781 (86.78)	269 (82.26)	158 (85.87)
≥ 1.50	268 (14.69)	203 (14.39)	119 (13.22)	58 (17.74)	26 (14.13)
Log(TG/HDL)					
< 0.50	1701 (93.26)	1317 (93.34)	846 (94.00)	302 (92.35)	169 (91.85)
≥ 0.50	123 (6.74)	94 (6.66)	54 (6.00)	25 (7.65)	15 (8.15)
LDL/HDL					
< 3.50	1658 (90.90)	1280 (90.72)	824 (91.56)	289 (88.38)	167 (90.76)
≥ 3.50	166 (9.10)	131 (9.28)	76 (8.44)	38 (11.62)	17 (9.24)
ApoB/ApoA-I					
< 1.00	1496 (82.02)	1165 (82.57)	761 (84.56)	258 (78.90)	146 (79.35)
≥ 1.00	328 (17.98)	246 (17.43)	139 (15.44)	69 (21.10)	38 (20.65)

Proportional odds ratios (ORs) with 95% confidence intervals for the association between the dichotomised serum biomarkers and BC severity at time of diagnosis are displayed in Table 3. Serum glucose (OR: 1.25; 95%CI: 0.92–1.70) and the ApoB/ApoA-1 ratio (OR: 1.31; 95%CI: 0.97.05–1.76) showed greater odds of being diagnosed with a more severe BC in women with perturbed serum levels of these biomarkers. Thus, for those women with serum glucose levels ≥5.60 mmol/l, the odds of severe BC versus the combined categories of moderate and good were 1.25 times greater, given that all of the remaining variables in the model were held constant. Similarly, because of the proportional odds assumption, the odds of the combined severe and moderate categories of BC versus good sees the same increase of 1.25 times greater, given that all of the remaining variables in the model were held constant. In a further analysis we stratified by interval time, but did not observe any consistent pattern (results not shown). Additional adjustment where interval time was included in the model as a continuous variable also did not change the above findings (results not shown).

**Table 3:** Proportional odds ratios (OR) and 95% confidence intervals (CI) for breast cancer severity from a multivariate model, including women from the AMORIS database and the Swedish Breast Cancer Registry between the years 1989–2011. Models were adjusted for age, parity, socioeconomic status, fasting status, total cholesterol, glucose, triglycerides, and interval time (except where stated otherwise)

	All BC w/severity (*N* = 1411)	Crude Model	Adjusted Model
	N(%)	OR (95%CI)	OR(95%CI)
Total Cholesterol (mmol/l)^a^			
Log TC		1.14 (0.66–1.97)	0.96 (0.51–1.83)
< 6.50	1040 (73.71)	1.00 (ref)	1.00 (ref)
≥ 6.50	371 (26.29)	1.19 (0.95–1.49)	1.08 (0.85–1.38)
Triglycerides (mmol/l)^b^			
Log TG		1.22 (1.00–1.50)	1.21 (0.96–1.53)
< 1.71	1237 (87.67)	1.00 (ref)	1.00 (ref)
≥ 1.71	174 (12.33)	1.13 (0.84–1.52)	1.00 (0.72–1.37)
HDL (mmol/l)			
Log HDL		0.66 (0.45–0.98)	0.73 (0.44–1.21)
≥ 1.03	1364 (96.67)	1.00 (ref)	1.00 (ref)
< 1.03	47 (3.33)	1.10 (0.63–1.90)	0.89 (0.47–1.67)
LDL (mmol/l)			
Log LDL		1.23 (0.87–1.74)	2.09 (0.92–4.78)
< 4.10	1032 (73.14)	1.00 (ref)	1.00 (ref)
≥ 4.10	379 (26.86)	1.12 (0.89–1.40)	1.07 (0.76–1.50)
ApoA (g/L)			
Log ApoA		0.49 (0.25–0.98)	0.56 (0.27–1.15)
≥ 1.05	1402 (99.36)	1.00 (ref)	1.00 (ref)
< 1.05	9 (0.64)	2.12 (0.67–6.69)	1.99 (0.62–6.35)
ApoB (g/L)			
Log ApoB		1.23 (0.82–1.85)	1.30 (0.60–2.81)
< 1.50	1208 (85.61)	1.00 (ref)	1.00 (ref)
≥ 1.50	203 (14.39)	1.25 (0.95–1.65)	1.22 (0.85–1.74)
Glucose (mmol/)^c^			
Log Glucose		1.76 (0.99–3.14)	1.63 (0.88–3.01)
< 5.60	1231 (87.24)	1.00 (ref)	1.00 (ref)
≥ 5.60	180 (12.76)	1.38 (1.04–1.84)	1.25 (0.92–1.70)
Log(TG/HDL)^b,c^			
Log TG/HDL		1.19 (1.02–1.39)	1.16 (0.99–1.38)
< 0.50	1317 (93.34)	1.00 (ref)	1.00 (ref)
≥ 0.50	94 (6.66)	1.32 (0.90–1.94)	1.16 (0.78–1.74)
LDL/HDL			
Log LDL/HDL		1.27 (1.00–1.62)	1.29 (0.93–1.78)
< 3.50	1280 (90.72)	1.00 (ref)	1.00 (ref)
≥ 3.50	131 (9.28)	1.22 (0.87–1.71)	1.10 (0.74–1.63)
ApoB/ApoA-1			
Log ApoB/ApoA-1		1.38 (0.98–1.95)	1.37 (0.88–2.15)
< 1.00	1165 (82.57)	1.00 (ref)	1.00 (ref)
≥ 1.00	246 (17.43)	1.36 (1.05–1.75)	1.31 (0.97–1.76)

^a^ Not adjusted for TC; ^b^ Not adjusted for TG; ^c^ Not adjusted for glucose

**Fig. 2:**
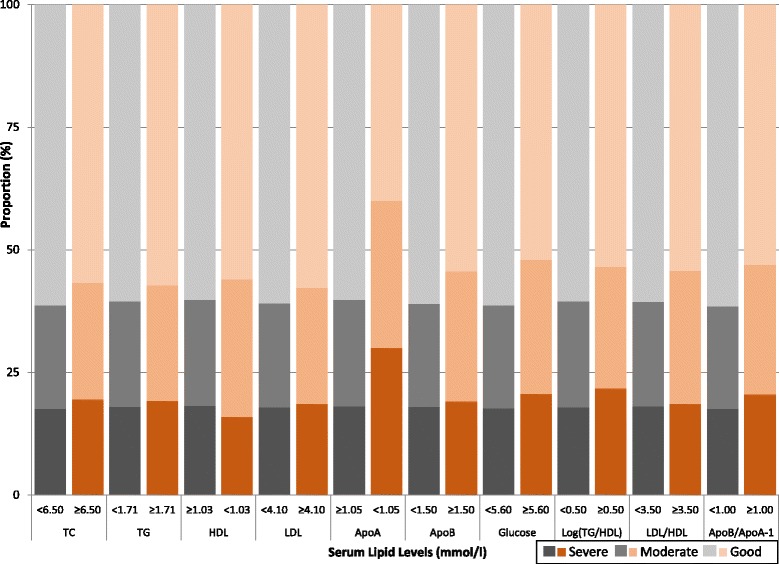
Proportion of breast cancer severities within each dichotomised serum biomarker

The proportion of severe and moderate BC was greater across all perturbed serum biomarker groups, with a statistical difference observed for the ApoB/ApoA-1 ratio (*p*-value =0.03) (Table 3). For example, for serum levels of ApoA-1, the proportion of moderate and severe BCs was greater in women with reduced levels of ApoA-1 (<1.05 mmol/l). In agreement with the proportional ORs, the proportion of women with severe and moderate BC was moderately greater in women with elevated serum glucose levels compared to those with normal glucose levels. Similarly the proportion of moderate and severe BCs was higher for those with elevated values for the ApoB/ApoA-1 ratio. The distribution of BC severity was graphically illustrated to further evaluate the associations observed with lipid levels (Fig. 2).

Finally, we tested for an interaction term between interval time (time between baseline measurement and BC diagnosis) and each serum biomarker, but did not identify any statistically significant effect modification by interval time (results not shown).

## Discussion

Only a weak positive association between both serum glucose and the ApoB/ApoA-1 ratio and the odds of a more severe BC at time of diagnosis was observed. For every serum biomarker studied, the overall proportion of moderate and severe BCs was greater amongst women with clinically abnormal values, compared to women with values within the normal clinical range. However, the differences in proportions were small. When investigating a possible modifying effect of the interval time between the measurement and BC diagnosis, no significant patterns were found.

Components of the MetS, particularly obesity, dyslipidemia, and diabetes have rapidly become health issues around the world, and have been independently associated with an increased risk of BC, especially postmenopausal BC [1, 4, 15]. Increased adiposity is associated with insulin resistance and dyslipidemia, both of which have been shown to increase BC risk [16, 42].

### The glucose metabolism

Based on epidemiological studies, it is thought that elevated serum glucose increases the risk of BC. For example, a Chinese cohort study found that women with abnormal glucose markers, measured several years prior to their diagnoses, had a BC prevalence ratio of 1.56 (95%CI: 1.21–2.00) [11]. However, a similar investigation into the significance of glucose in the risk of developing BC in the Framingham heart study-offspring cohort found only a non-significant increased risk [39].

The rise in serum glucose levels, which accompanies insulin resistance, has also been cited to result in a worse prognosis, but only in postmenopausal BC [37, 49, 53]. While hyperglycaemia has been reported as a possible mechanism, it is not thought to be the primary candidate, with insulin-like growth factor 1 (IGF-1) believed to have a greater influence [53]. Here we saw a weak trend between elevated serum glucose levels and a poor BC prognosis, although the risk was not particularly large. This could potentially be explained by hyperglycaemia being a symptom of hyperinsulinaemia (as a consequence of insulin resistance), and perhaps a stronger association would be have been found if IGF-1 were investigated directly.

### The lipid metabolism

Previously in AMORIS we found weak evidence that abnormal serum lipid profiles may be involved in the risk of developing BC [35]. However, existing literature has cited associations between other components of the lipid metabolism. For example, increased triglycerides have been found to be associated with BC risk [13] as well as low HDL levels [22]. The latter was confirmed in another study, which also noted an inverse association between BC risk and serum levels of TC and ApoA-1 [25]. On the contrary, although no comment was made on BC severity, the MetS has been proposed as a risk factor for BC in postmenopausal women only [2, 12]. However, in our current study, we did not observe associations with regards to BC severity for these serum lipid markers. This contrast with our previous AMORIS studies may thus be due to the different epidemiological framework studied: the etiologic (i.e. BC versus non-BC) versus the prognostic (i.e. breast cancer patient only) setting.

Additionally, some studies suggest that ApoA-1 is associated with a decreased BC risk [25, 38]. Recent studies also raised the question as to whether the ratio of ApoB to ApoA-1 may be an independent risk predictor [43, 44, 50]. Thus, we incorporated these measurements into our investigation into BC severity. This ratio reflects the balance between all atherogenic ApoB-containing lipoprotein particles and ApoA-I, indicating athero-protective particles [44, 45]. Our observation of a weak trend between the apoB/apoA-1 ratio and BC severity may reflect characteristics of the MetS such as abdominal obesity playing a role in BC development – however, experimental evidence is needed to support this hypothesis.

It was of interest to study the interval time between baseline measurement and BC diagnosis, as one could assume that those with more severe BC may have had their disease for a longer time, which would reflect in reverse causation: the undiagnosed BC may be associated with perturbed serum lipid levels. Furthermore, even among women with less severe BC it is possible that lipid levels in the short time prior to diagnosis may already be affected by the process of carcinogenesis [21]. However, in our study we did not find any interaction with interval time.

Measurement of these biomarkers at an average of 11 years prior to the diagnosis of breast cancer has both benefits and disadvantages. The true distribution of induction and promotion times for breast cancer is not known. It is likely that the time window of exposure in this study reflects induction and unlikely that reverse causation by the presence of an established cancer is playing a role. However, it is plausible that the lipid metabolism changed substantially in the period between measurement and diagnosis due to lifestyle changes. Alternatively, it could be possible that the individual develops another co-morbidity (e.g., coronary heart disease, or diabetes) which acts as a competing risk, and may result in death prior to diagnosis of a cancer ([40]. However, the AMORIS cohort is still relatively young and competing risks due to cardiovascular and metabolic disease would be a threat mainly in follow-up of women aged 70 and older. To take into account these potential effects, it would be of interest to have a study setting where measurements are repeated over time – hence, it is a limitation that our study was only based on single measurements.

A major strength of the AMORIS database is its large size, and prospective blood profile measurements for all individuals, measured at the same laboratory (CALAB). The small number of women with abnormal values for certain biomarkers may have caused insufficient statistical power to detect significant associations. However, when using the continuous log value of these biomarkers none of the results changed. Additionally, we included CCI in the models to account for potential confounding by diabetes, BMI, smoking habits, diet, or hypertension. Previously, a limitation of the AMORIS database was the lack of information on tumour characteristics, which has now been accommodated by the recent linkage to the Breast Cancer Quality Register. We did not have data on menopausal status, but age at time of diagnosis was included in our definition of BC severity and an additional stratification by age was conducted.

## Conclusion

High levels of serum glucose and the ApoB/ApoA-1 ratio were observed to be only modestly associated with higher odds of having more severe BC. Thus, in spite of the size and great detail of the data in AMORIS, we only found a modest positive association between serum levels of glucose, apoB/ApoA-1 and BC severity, suggesting that these factors are not the main players in the link between obesity and BC aggressiveness.

## Additional files

Additional file 1: **Figure S1.** Overview of the study cohort. (PPTX 32 kb)

Additional file 2: **Figure S2.** A Kaplan-Meier curve for survival in all breast cancer patients in the AMORIS cohort, by severity status (*n* = 12,537) assessed at diagnosis. (PPTX 45 kb)

## Abbreviations

95%CI: 95% Confidence Interval
AMORIS: Apolipoprotein Mortality Risk
Apo: Apolipoprotein
BC: Breast cancer
CALAB: Central Automation Laboratory
CCI: Charlson Comorbidity Index
HDL: High density lipoprotein
HR: Hazard Ratio
IGF: Insulin-like growth factors (IGF)
LDL: Low density lipoprotein
TC: Total cholesterol
TG: Triglycerides
