# Supplementary material for: Glucose and lipoprotein biomarkers and breast cancer severity using data from the Swedish AMORIS cohort
Source: BMC Cancer. 2017 Apr 4;17:246. doi: 10.1186/s12885-017-3232-6 (PMC5381045; doi:10.1186/s12885-017-3232-6)
Supplement: Supplementary file 1 — Figure S1. Overview of the study cohort. (PPTX 32 kb) [file 12885_2017_3232_MOESM1_ESM.pptx]

## Slide 1
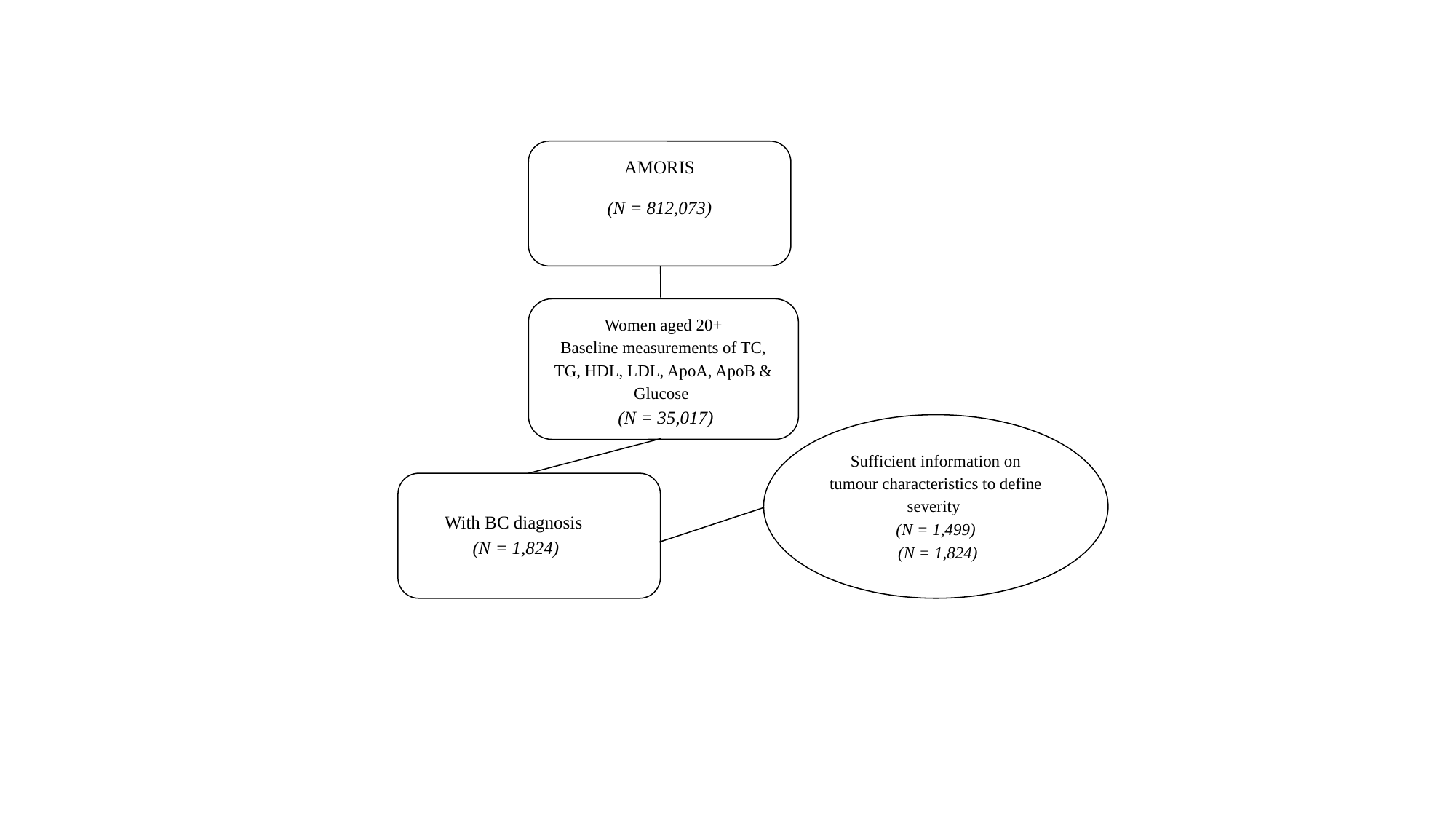

AMORIS
(N = 812,073)
Women aged 20+
Baseline measurements of TC, TG, HDL, LDL, ApoA, ApoB & Glucose
 (N = 35,017)
Sufficient information on tumour characteristics to define severity
(N = 1,499)
 (N = 1,824)
With BC diagnosis
 (N = 1,824)
