# Supplementary figures and images for: Glucose and lipoprotein biomarkers and breast cancer severity using data from the Swedish AMORIS cohort
Source: BMC Cancer. 2017 Apr 4;17:246. doi: 10.1186/s12885-017-3232-6 (PMC5381045; doi:10.1186/s12885-017-3232-6)

## Slide 1
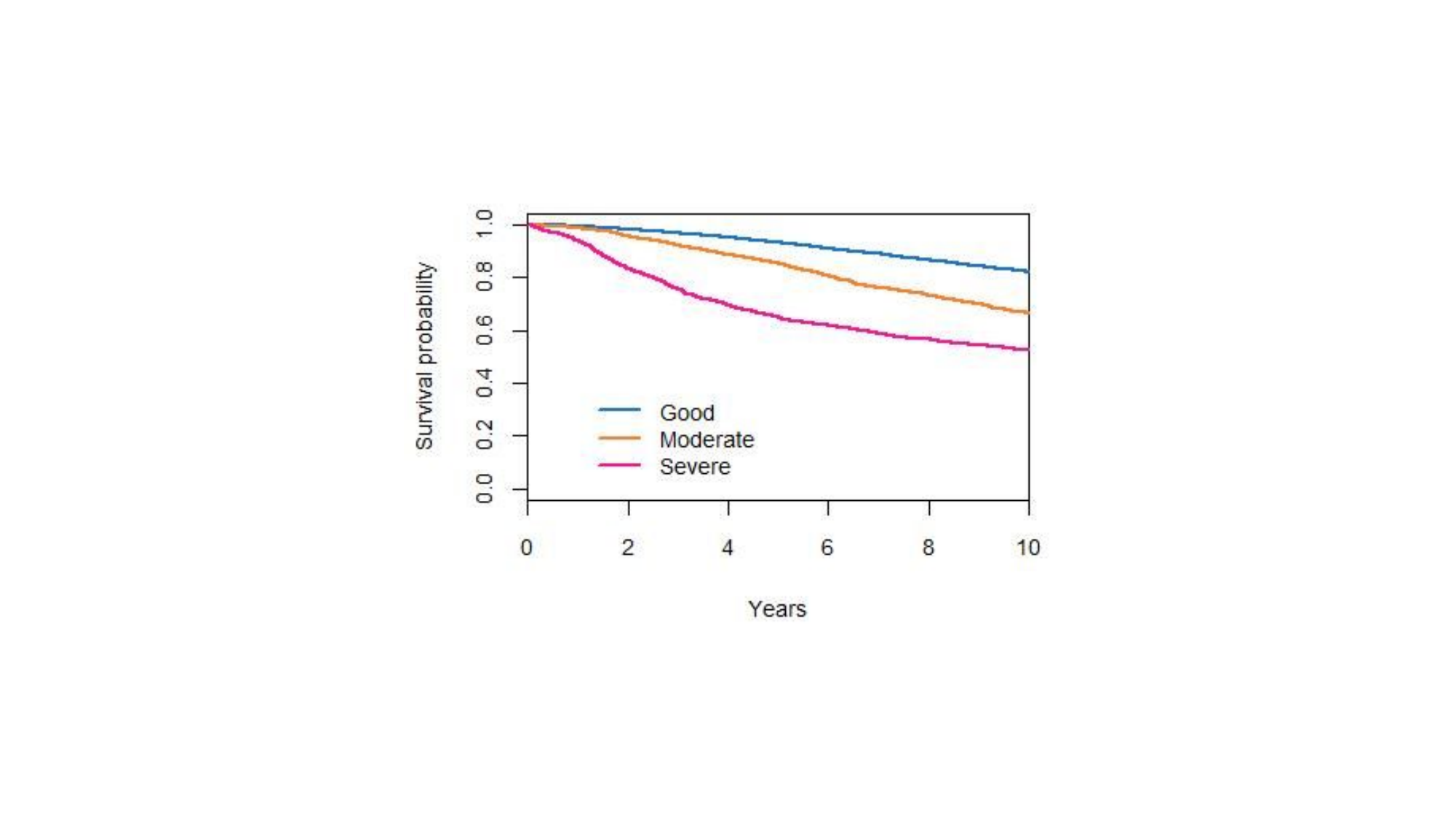

Supplement: Supplementary file 2 — Figure S2. A Kaplan-Meier curve for survival in all breast cancer patients in the AMORIS cohort, by severity status (n = 12,537) assessed at diagnosis. (PPTX 45 kb) [file 12885_2017_3232_MOESM2_ESM.pptx]
